# Discoidin Domain Receptors 1 Inhibition Alleviates Osteoarthritis via Enhancing Autophagy

**DOI:** 10.3390/ijms21196991

**Published:** 2020-09-23

**Authors:** Hsin-Chaio Chou, Chung-Hwan Chen, Liang-Yin Chou, Tsung-Lin Cheng, Lin Kang, Shu-Chun Chuang, Yi-Shan Lin, Mei-Ling Ho, Yan-Hsiung Wang, Sung-Yen Lin, Chau-Zen Wang

**Affiliations:** 1 Graduate Institute of Medicine, College of Medicine, Kaohsiung Medical University, Kaohsiung 80708, Taiwan; rna.studio2014@gmail.com (H.-C.C.); laining59@gmail.com (L.-Y.C.); homelin@kmu.edu.tw (M.-L.H.); 2 Orthopaedic Research Center, Kaohsiung Medical University, Kaohsiung 80708, Taiwan; hwan@kmu.edu.tw (C.-H.C.); junglecc@gmail.com (T.-L.C.); hawayana@gmail.com (S.-C.C.); 327lin@gmail.com (Y.-S.L.); yhwang@kmu.edu.tw (Y.-H.W.); 3 Regeneration Medicine and Cell Therapy Research Center, Kaohsiung Medical University, Kaohsiung 80708, Taiwan; 4 Departments of Orthopedics, Kaohsiung Municipal Ta-Tung Hospital, Kaohsiung 80145, Taiwan; 5 Institute of Medical Science and Technology, National Sun Yat-Sen University, Kaohsiung 80424, Taiwan; 6 Division of Adult Reconstruction Surgery, Department of Orthopedics, Kaohsiung Medical University Hospital, Kaohsiung Medical University, Kaohsiung 80708, Taiwan; 7 Department of Physiology, College of Medicine, Kaohsiung Medical University, Kaohsiung 80708, Taiwan; 8 Department of Obstetrics and Gynecology, National Cheng Kung University Hospital, College of Medicine, National Cheng Kung University, Tainan 70101, Taiwan; kanglin@mail.ncku.edu.tw; 9 Department of Orthopedics, College of Medicine, Kaohsiung Medical University, Kaohsiung 80708, Taiwan; 10 Department of Marine Biotechnology and Resources, National Sun Yat-Sen University, Kaohsiung 80424, Taiwan; 11 School of Dentistry, College of Dental Medicine Kaohsiung Medical University, Kaohsiung 80708, Taiwan; 12 Department of Medical Research, Kaohsiung Medical University Hospital, Kaohsiung 80708, Taiwan

**Keywords:** discoidin domain receptors 1 (Ddr1), osteoarthritis (OA), autophagy, apoptosis, terminal differentiation

## Abstract

We recently reported that the chondrocyte-specific knockout of discoidin domain receptors 1 (Ddr1) delayed endochondral ossification (EO) in the growth plate by reducing the chondrocyte hypertrophic terminal differentiation, and apoptosis. The biologic and phenotypic changes in chondrocytes in the articular cartilage with osteoarthritis (OA) are similar to the phenomena observed in the process of EO. Additionally, autophagy can promote chondrocyte survival and prevent articular cartilage from degradation in OA. On this basis, we explored the effect of Ddr1 inhibition on OA prevention and further investigated the roles of autophagy in treating OA with a Ddr1 inhibitor (7 rh). The anterior cruciate ligament transection (ACLT)–OA model was used to investigate the role of 7 rh in vivo. Forty 8-week-old mice were randomly assigned to four groups, including the sham group, ACLT group, and two treated groups (ACLT with 7 rh 6.9 nM or 13.8 nM). According to the study design, normal saline or 7 rh were intra-articular (IA) injected into studied knees 3 times per week for 2 weeks and then once per week for 4 weeks. The results showed that 7 rh treatment significantly improved the functional performances (the weight-bearing ability and the running endurance), decreased cartilage degradation, and also reduced the terminal differentiation markers (collagen type X, Indian hedgehog, and matrix metalloproteinase 13). Moreover, 7 rh decreased chondrocyte apoptosis by regulating chondrocyte autophagy through reducing the expression of the mammalian target of rapamycin and enhancing the light chain 3 and beclin-1 expression. These results demonstrated that the IA injection of 7 rh could reduce the chondrocyte apoptosis and promote chondrocyte autophagy, leading to the attenuation of cartilage degradation. Our observations suggested that the IA injection of 7 rh could represent a potential disease-modifying therapy to prevention OA progression.

## 1. Introduction

Osteoarthritis (OA) is the most prevalent joint disease worldwide and also the leading cause of disability in aged patients. Because of aging and increasing obesity in the population, the prevalence of OA is rapidly increased. The current pharmacological therapy for OA was focused on symptomatic relief. Despite some promising disease-modifying therapy undergoing clinical trials, there is no currently effective therapy to prevent the progressive destruction of articular cartilage [1].

Chondrocyte death is the key pathogenesis of OA. The articular chondrocyte is the only resident cell in cartilage and is responsible for maintaining the equilibrium of the extracellular matrix. The compromising of chondrocyte survival leads to cartilage degradation; as a result, the prevention of chondrocyte death is an important research direction for OA prevention. The morphologic changes in chondrocytes in OA are resembling those happening in the growth plates, where the chondrocytes undergo hypertrophic terminal differentiation, featuring chondrocyte hypertrophic change, mineralization, and eventually apoptosis [2]. Recent research on parathyroid hormone 1-34 (PTH 1-34) in OA has indicated that the treatment can inhibit the apoptosis of chondrocytes in the growth plate, which can also be used to inhibit the apoptosis of chondrocyte in osteoarthritic articular cartilage, thereby reducing cartilage degradation [3,4,5].

Discoidin domain receptors (Ddr) are a group of the transmembrane receptors that triggers a ligand-induced kinase activation and receptor autophosphorylation after biding collagen and then to regulate cell migration, proliferation, differentiation, and cell survival [6]. Ddr1 and Ddr2 are two major types of Ddrs with similar amino acid sequences and both are expressed in the cartilage. The biological function of Ddrs has been widely investigated by gene knockout mice. Although the physiologic role of two Ddrs is different, the global Ddr1 or Ddr2 ablated mice all present with dwarfism [7]. The Ddr2 ablation reduces the chondrocyte proliferation and thus decreases bone growth; however, the reasons for dwarfism in Ddr1 ablation mice remain unclear. Our recent study using chondrocyte-specific Ddr1 knockout mice demonstrated that the Ddr1 ablation resulted in a decreased chondrocyte proliferation, terminal differentiation, and apoptosis in growth plates and caused a delayed EO [8]. Our findings indicated that Ddr1 is an imperative regulator of EO in the growth plate and maybe also a potential target for OA treatment.

Autophagy maintains the homeostatic equilibrium to cartilage following injury and is reported to be a cellular self-protection mechanism of normal cartilage. Previous researchers demonstrated that the deregulation of autophagy predisposed to the pathogenesis of OA and the activation of autophagy could protect articular cartilage from OA [9]. The mammalian target of rapamycin (mTOR) is a serine/threonine protein kinase regulating signal transduction during autophagy. A previous study showed that the cartilage-specific deletion of mTOR upregulated the autophagy signaling and reduced the articular degradation, chondrocyte apoptosis, and synovial fibrosis in surgical-induced OA mice [10]. Similarly, rapamycin (an inhibitor of mTOR) enhanced the activation of autophagy, maintained the cartilage cellularity, and reduced the severity of surgical-induced OA [11]. Although we do not fully understand the relationship between chondrocyte survival and autophagy, accumulating evidence suggests that the restoration autophagy of senescent chondrocyte can restore the reparative capacity and may be an effective therapeutic approach for OA [12]. 

While the inhibiting of Ddr1 can decrease chondrocyte apoptosis in the growth plate, it has yet to be demonstrated whether this phenomenon may occur in the articular cartilage and the inhibition expression of Ddr1 can protect against cartilage degradation in experimental-induced OA animal models. Ddr1 inhibition can reduce chondrocyte terminal differentiation and apoptosis in the growth plate, but whether autophagy contributes to the treatment of Ddr1 suppression on ameliorating OA progression remains unclear. The object of this study was to test the hypothesis that the inhibition of Ddr1 expression by intra-articular (IA) injection of Ddr1 inhibitor (7 rh) can reduce the OA progression in anterior cruciate ligament transection (ACLT)-induced OA mice. Specifically, we aimed to determine whether (1) IA of 7 rh can delay the articular cartilage degradation in histology and also improve knee function after OA induction; (2) IA of 7 rh can reduce the chondrocyte terminal differentiation and apoptosis; (3) the effects of 7 rh on prevention chondrocyte apoptosis are through the regulation of autophagy in chondrocytes.

## 2. Results

### 2.1. Low Cytotoxicity of 7 rh on Human Articular Chondrocytes Viability

**Figure 1:**
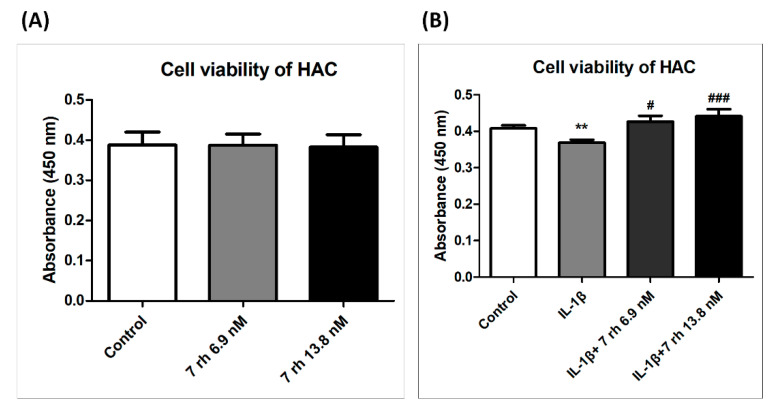
The cytotoxic effects of 7 rh on human articular chondrocytes (HAC). (**A**) The cell viability of HAC that was treated with 6.9 nM and 13.8 nM of 7 rh for 48 h was detected by CCK8 assay. (**B**) The chondrocytes were co-cultured with IL-1β and 7 rh (6.8 nM and 13.9 nM) for 48 h. The cell viability was measured by CCK8 assay. All experiments were performed in quadruplicate and repeated three times with similar results. (** *p* < 0.01 versus the control group; ^#^ *p* < 0.05, and ^###^ *p* < 0.001 versus the IL-1β-treated group).

A pilot study was conducted to choose the optimal concentrations of 7 rh for this experiment. The cytotoxicity of 7 rh on HAC was assessed by CCK8 assay. IC50 (6.9 nM) and IC100 (13.8 nM) of 7 rh were assessed to determine the cytotoxicity. Cell viability assay exhibited that the drug dosage in our study was low cell cytotoxicity to chondrocyte (Figure 1). Therefore, we selected the concentrations of 6.9 nM and 13.8 nM for the subsequent experiments. Additionally, the effects of 7 rh on IL-1β-stimulated HAC were assessed. As shown in Figure 1B, the results showed that the cell viability was suppressed after IL-1β stimulation and 7 rh could significantly prevent the inhibitory effects of IL-1β on cell viability at both selected concentrations (*p* < 0.05 in 6.9 nM and *p* < 0.001 in 13.8 nM).

### 2.2. The IA Administration of 7 rh Improves the Weight Distribution Ability and Running Endurance after OA-Induction

**Figure 2:**
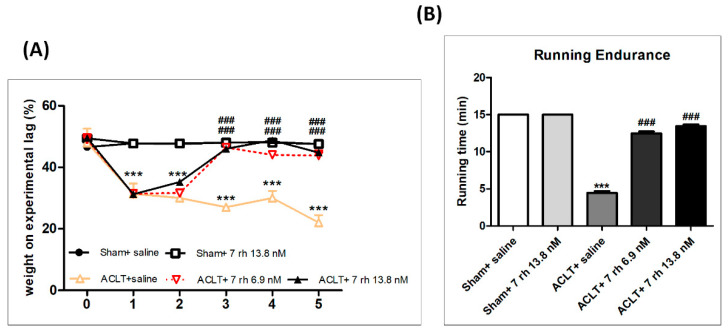
Treatment with 7 rh promoted knee function after anterior cruciate ligament transection (ACLT). (**A**) Weight-bearing test. There were no obvious influences in both of the sham control groups (sham+ saline and sham+ 7 rh 13.8 nM) during the treating courses. The weight-bearing ability of the studied limb was significantly reduced after osteoarthritis (OA) induction (*p* < 0.001). Mice in the OA + 7 rh group could bear significantly more weight since the third week (*p* < 0.001 at both concentrations) and thereafter until the end of the study (all *p* < 0.001). (**B**) Treadmill running test. The mice in the ACLT-OA group could endure less time in gait occurred than those in the sham control groups (*p* < 0.001) at 5 weeks. In both treating groups, the mice significantly increased their running endurance in the treadmill test (all *p* < 0.001) with no significant difference from mice in the sham control groups. (*** *p* < 0.001 versus the sham control groups; ^###^ *p* < 0.001 versus the ACLT group).

The weight distribution test and running endurance assessment were used to evaluate the functional performance of knee joints after OA induction with or without 7 rh treatment. During the treatment course, we did not observe an apparent change in weight-bearing ability of the studied limb in both of the sham control groups (sham+ saline and sham+ 7 rh 13.8 nM), but a significantly reduced ability to bear weight in ACLT mice (*p* < 0.001 compared with the sham control groups). In contrast, the mice in 7 rh-treated groups could bear approximately 50% of their body weight since the third week, and the effects persisted to the residual treatment course (Figure 2A).

The running endurance is an indicator of knee function improvement. As the results of the weight distribution test, the running endurance was significantly reduced after OA induction. The mice in both sham control groups could finish the 15 min running test, whereas the mice in the ACLT group could only endure 4.5 ± 0.8 min at 5 weeks. Notably, 7 rh significantly increased the mice’ endurance levels to 12.5 ± 0.8 min in 6.9 nM 7 rh group and 13.5 ± 0.8 min in the 13.8 nM 7 rh group, respectively, at 5 weeks, which is not a significant difference compared to the sham control groups (Figure 2B).

### 2.3. The IA Administration of 7 rh Slows the Articular Cartilage Degradation

**Figure 3:**
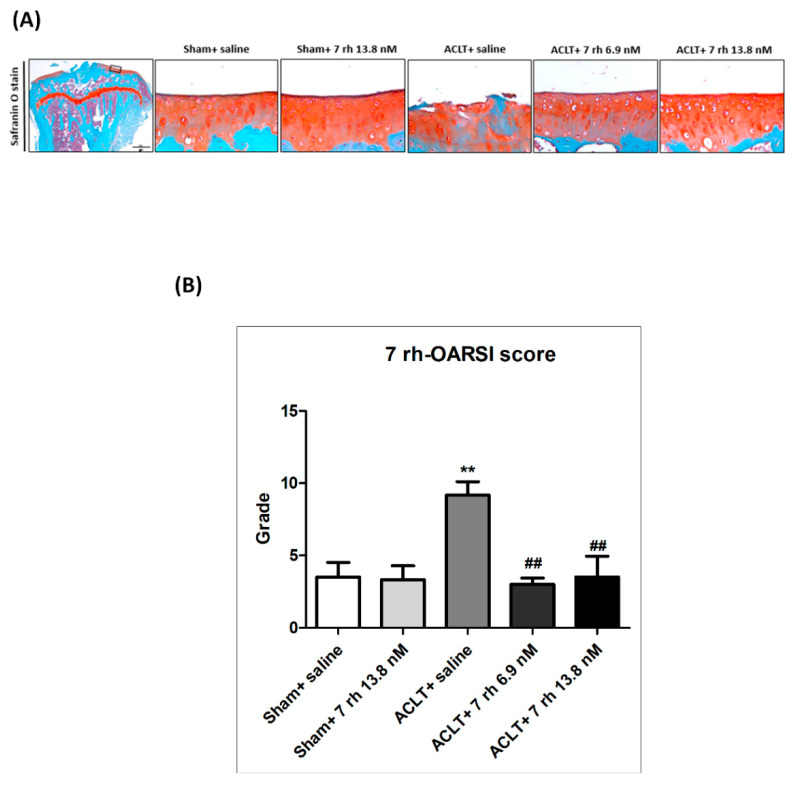
Intra-articular injection of 7 rh attenuated cartilage degradation in anterior cruciate ligament transection (ACLT)-induced osteoarthritis (OA). (**A**) The representative Safranin O-Fast Green stained articular cartilages micrographs of the proximal tibia from the right knee joints of mice in the sham control groups (sham+ saline and sham+ 7 rh 13.8 nM), ACLT, and two 7 rh treated groups. (**B**) Osteoarthritis Research Society International (OARSI) scores of articular cartilage at 5 weeks after ACLT surgery. (** *p* < 0.01 versus the sham control groups; ^##^ *p* < 0.01 versus the ACLT group).

To evaluate the efficacy of 7 rh on ACLT-induced OA progression, the loss of GAG, and the structural integrity of the articular cartilage were examined by the Safranin O-Fast Green staining and OARSI histology grading scores. As shown in Figure 3A, there was no obvious structure destruction or GAG loss identified in the sham-operated knees that were treated either with normal saline or 7 rh 13.8 nM. In contrast, we observed apparent damages to articular cartilage with a marked loss of proteoglycans in the ACLT mice at 5 weeks, indicating the successful induction of OA. The IA injection of 7 rh (6.9 nM and 13.8 nM) attenuated the cartilage degeneration and reduced the GAG loss after ACLT compared with the sham-operated knees. These results were further confirmed by the OARSI scores. The articular cartilage in the sham-operated knees did not exhibit obvious OA pathological changes in the articular cartilage and had an average OARSI score of 3.2 ± 0.9 in saline group and 3.3 ±1.0 in 7 rh 13.8 nM group (Figure 3B). The cartilage in the ACLT-OA knees exhibited significant GAG loss, cartilage erosion, and chondrocyte clustering with a mean OARSI score 9.2 ± 0.9, which was significantly higher compared to the sham-operated knees (*p* < 0.01). In contrast, the cartilage in the 7 rh-treated mice revealed a less cartilage fibrillation and proteoglycans loss and the mean OARSI scores (4 ± 0.9 in 6.9 nM of 7 rh, and 2.8 ± 1 in 13.8 nM of 7 rh) were also significantly lower compared to ACLT-OA mice (*p* < 0.01 in both concentrations).

### 2.4. The IA Administration of 7 rh Decreases the Expression of Hypertrophic Markers (Col X, MMP13, and IHH) after OA Induction

**Figure 4:**
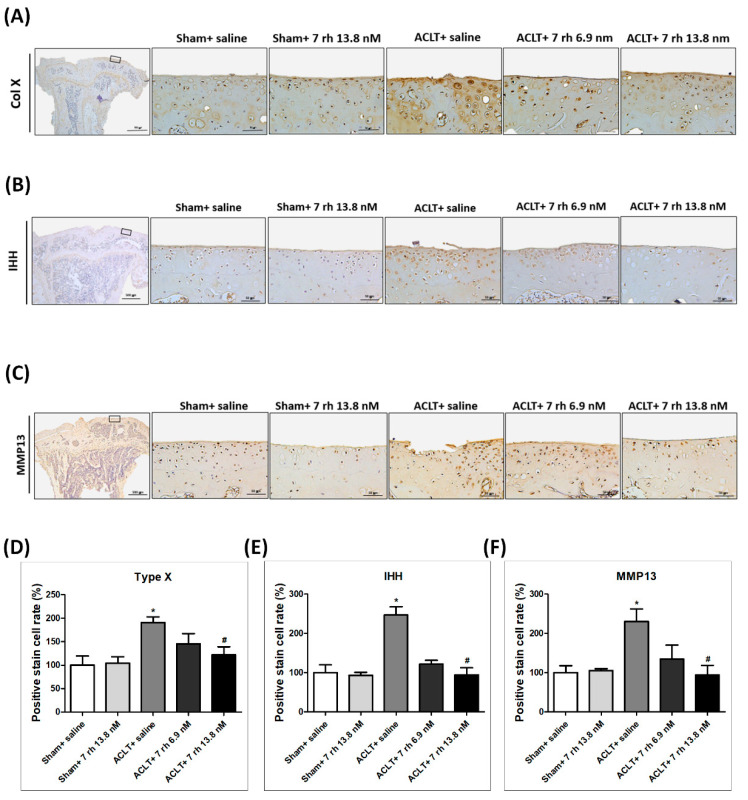
Intra-articular injections of 7 rh reduced the chondrocyte hypertrophic differentiation in anterior cruciate ligament transection (ACLT)-induced osteoarthritis (OA) cartilage. (**A**) The representative micrographs of immunolocalized type X collagen (Col X) in articular cartilage of the sham control groups (sham+ saline and sham+ 7 rh 13.8 nM), ACLT, and two 7 rh treating groups. (**B**) The representative micrographs of immunolocalized Indian hedgehog (IHH). (**C**) The representative micrographs of immunolocalized matrix metallopeptidase 13 (MMP13). (**D**) Quantitative analysis of the immunohistochemically (IHC) staining of Col X. (**E**) Quantitative analysis of the IHC staining of IHH. (**F**) Quantitative analysis of the immunohistochemical IHC of MMP13. In quantitative analysis, each bar represents the mean±SEM of 12 samples in each group. (* *p* < 0.05 versus the sham control groups; ^#^ *p* < 0.05 versus the ACLT group).

IHC staining was performed to investigate whether 7 rh alleviated OA progression by reducing the chondrocyte hypertrophic differentiation. The representative images of IHC staining of chondrocyte hypertrophic markers, including Col X, IHH, and MMP13 at 5 weeks, are shown in Figure 4A–C and the results of the quantitative analysis are shown in Figure 4D–F. Col X, IHH, and MMP13 were well-established markers for detecting the chondrocyte during the hypertrophic differentiation. The density of immunolocalized Col X was significantly increased in the ACLT group, and the treatment of 7 rh (13.8 nM) in ACLT mice at 5 weeks significantly inhibited the expression of Col X (Figure 4A,D). As shown in Figure 4B,E, the levels of IHH expression also increased after OA induction and reduced significantly in the 7 rh-treated mice (13.8 nM group) compared to ACLT-OA mice at 5 weeks. As shown in Figure 4C,F, the immunolocalized MMP13 protein was predominantly found in the articular chondrocytes in the ACLT-OA mice and the expressions were decreased significantly after 7 rh treatment (13.8 nM group). These results revealed that chondrocytes undergo terminal differentiation after OA induction, but 7 rh (13.8 nM) can prevent chondrocytes from terminal differentiation.

### 2.5. The IA Administration of 7 rh Decreases Chondrocyte Apoptosis

**Figure 5:**
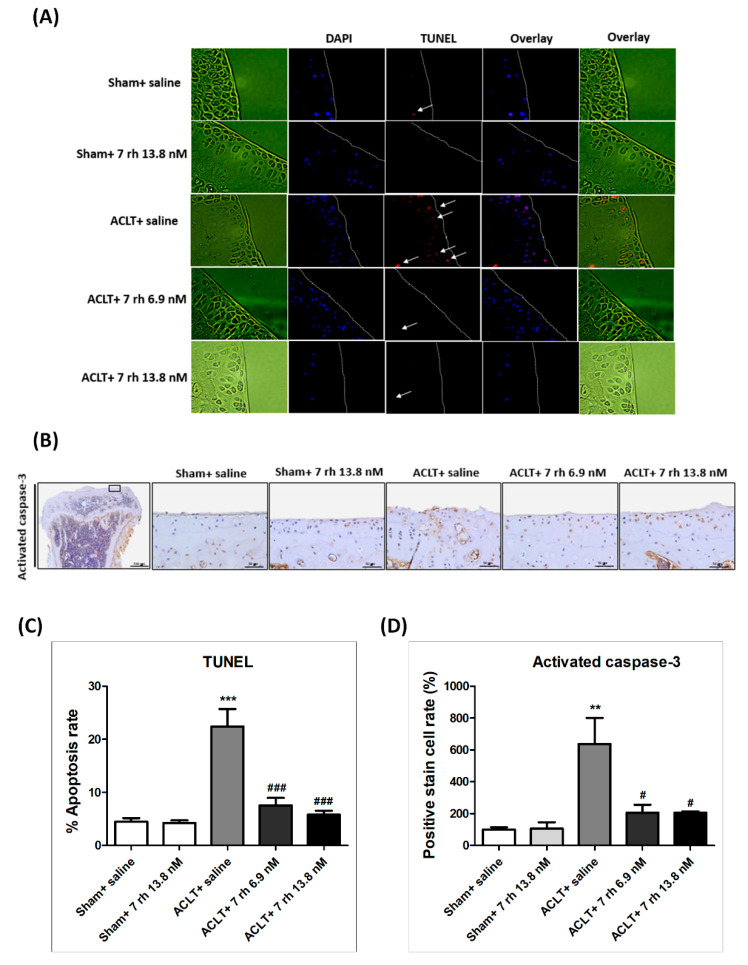
Effect of 7 rh on chondrocyte apoptosis in articular cartilage. (**A**) TUNEL staining in the articular cartilages of the sham control groups (sham+ saline and sham+ 7 rh 13.8 nM), ACLT, and two 7 rh treating groups. The arrows mean positive stain of TUNEL. (**B**) The representative micrographs of immunolocalized activated caspase 3. (**C**) Quantitative analysis of the apoptotic rate. (**D**) Quantitative analysis of the immunohistochemical staining of activated caspase 3. Each bar represents the mean±SEM of 12 samples in each group. (** *p* < 0.01 versus the sham control groups; *** *p* < 0.001 versus the sham control groups; ^#^ *p* < 0.05 versus the ACLT group; ^###^ *p* < 0.001 versus the ACLT group).

The results of TUNEL staining exhibited that 7 rh treatment prevented cell apoptosis in the ACLT-OA cartilage (Figure 5A). The apoptotic rate of chondrocytes in the articular cartilage of the ACLT-OA mice (22.4 ± 3.3%) was significantly more increased than in the sham + saline group (4.5 ± 0.7%, *p* < 0.001) and in the sham + 7 rh 13.8 nM group (1.0 ± 0.5%, *p* < 0.001). After 7 rh treatment at 5 weeks, the apoptotic rate of chondrocytes in the 7 rh-treated articular cartilage was significantly reduced to 7.6 ± 1.4% in the 7 rh 6.9 nM group and 5.8 ± 0.8% in the 7 rh 13.8 nM group, respectively (Figure 5C). 

Caspase-3 is considered as the primary executioner for apoptosis; therefore, we used IHC staining of activated caspase-3 for the analysis of apoptosis activity. The results revealed that activated caspase-3 protein expression in the ACLT group was significantly increased compared with the sham control groups, but was significantly decreased in the OA-induced mice treated with 7 rh (both 6.9 nM and 13.8 nM) at 5 weeks (Figure 5B,D). These results showed that 7 rh significantly reduced chondrocyte apoptosis in ACLT mice.

### 2.6. The IA Administration of 7 rh Decreases mTOR Expression and Increases LC3 and Beclin 1 Expression

**Figure 6:**
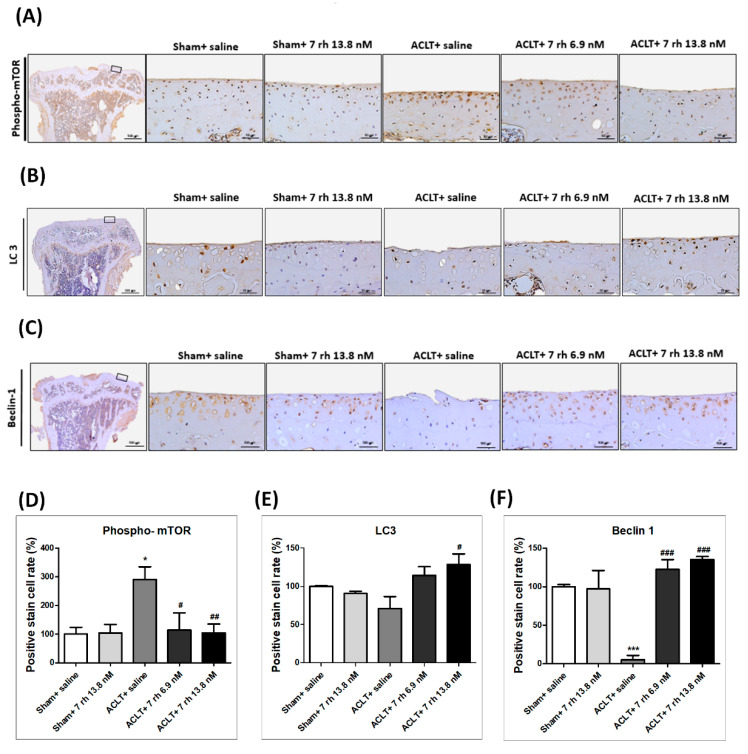
Effect of 7 rh mediated enhanced autophagy-related proteins in articular cartilage. (**A**) The representative micrographs of immunolocalized phosphorylation of mechanistic target of rapamycin (phospho-mTOR) in articular cartilage of the sham control groups (sham+ saline and sham+ 7 rh 13.8 nM), ACLT, and two 7 rh treated groups. (**B**) The representative micrographs of immunolocalized light chain 3 (LC3). (**C**) The representative micrographs of immunolocalized beclin-1. (**D**) Quantitative analysis of the immunohistochemical (IHC) staining of phospho-mTOR. (**E**) Quantitative analysis of the IHC staining of LC3. (**F**) Quantitative analysis of the IHC immunohistochemical staining of beclin-1. In quantitative analysis, each bar represents the mean±SE of 12 samples in each group. (* *p* < 0.05 versus the sham control groups; *** *p* < 0.001 versus the sham control groups; ^#^ *p* < 0.05 versus the ACLT group; ^##^ *p* < 0.005 versus the ACLT-OA group; ^###^ *p* < 0.001 versus the ACLT group).

To clarify if 7 rh treatment can rescue the autophagy effect in the ACLT-OA model, we examined the expression of autophagy markers, including mTOR, LC3 and beclin-1 in the articular cartilage (Figure 6). The results of IHC staining showed that the autophagy dysfunction in response to ACLT-OA induction was rescued by 7 rh. The expression of phosphorylated mTOR in the 7 rh treated group was significantly lower than that in the ACLT group (*p* < 0.05 in 7 rh 6.9 nM group and *p* < 0.01 in 7 rh 13.8 nM group). Meanwhile, the density of LC3 and beclin 1 in the cartilage of the 7 rh treated group was significantly higher than that in the ACLT group at 5 weeks (*p* < 0.05 in LC3 and *p* < 0.001 in beclin 1).

## 3. Discussion

The results of the current study are the first to show the central role of the Ddr1 inhibition in ameliorating OA progression in the ACLT induced OA model. By using a Ddr1 inhibitor (7 rh), we significantly slowed the articular cartilage degradation, improved the weight-bearing ability, and promoted the running endurance through treadmill tests at 5 weeks after ACLT. Histologically, the reduced expression of chondrocyte hypertrophic markers indicated the inhibition of chondrocyte terminal differentiation. Furthermore, 7 rh reduced the chondrocyte hypertrophic differentiation and apoptosis by enhancing autophagy.

Previous studies have indicated the similar biological behaviors of articular chondrocytes in OA progression with the chondrocytes of the growth plate in EO [2]. In the OA status, the articular chondrocytes become hypertrophic, with changes accompanied by the overexpression of the hypertrophic markers, including alkaline phosphatase, Col X, and MMP13 and subsequent chondrocyte mineralization and apoptosis, which is similar with the phenotype changes in EO [13]. It was already known that the chondrocyte differentiation and EO in the growth plate were regulated by the parathyroid hormone-related protein (PTHrP)-IHH feedback loop [14]. PTH 1–34, sharing the same receptors with PTHrP, was also reported to suppress the expression of Col X and IHH of hypertrophic chondrocyte in the fetal bovine growth plates [15]. In our previous study, we showed that IA injection of the PTH 1–34 at 3–5 weeks significantly attenuated the loss of proteoglycans and type II collagen, suppressed the expression of Col X, and reduced chondrocyte apoptosis in papain-induced OA in rats [3]. Similar results have also been reported in the systemic administration of PTH 1-34 [5] and in both surgical induced and spontaneous OA models [4]. These studies proved the concepts that treatments that can inhibit chondrocyte terminal differentiation in the growth plate and can also suppress chondrocyte apoptosis in articular cartilage and then inhibit the progression of OA.

Our recent research, investigating the role of Ddr1 in the regulation of EO, has indicated that the chondrocyte-specific Ddr1 knockout can delay the EO and accompany decreased chondrocyte proliferation, terminal differentiation, and apoptosis in growth plates of mice [8]. Accordingly, we hypothesized that the Ddr1 inhibitor (7 rh) may maintain the survival of articular chondrocytes and reduce the progression of OA. To test this hypothesis, we examined the efficacy of the IA injection of 7 rh on reducing the cartilage degeneration and inhibition of the chondrocyte apoptosis in the ACLT-induced OA model in mice. Our results indicated that the IA injection of 7 rh improved the function of the OA joint and reduced the OARSI score. We also found that 7 rh can decrease chondrocyte hypertrophic differentiation by inhibiting the expression of Col X, MMP13, and IHH. IHH signaling is not only essential for regulating normal chondrocyte proliferation and differentiation in the growth plate but also important in modulating chondrocyte terminal differentiation in OA cartilage. Lin A.C. et al. used genetically modified mice to exam the role of IHH signaling in OA chondrocytes and demonstrated that the expression of IHH was up-regulated by OA and was closely related to the severity of OA [13]. Moreover, the pharmacological inhibition of IHH signaling could decrease the hypertrophic differentiation of chondrocytes and ameliorate OA severity [13,14]. Accordingly, we reputed that the inhibition of the up-regulation of IHH signaling in OA plays a pivotal role in suppressing the degradation of cartilage. 

The hypertrophy-like changes in chondrocytes secreted a number of proteins that are involved in tissue remodeling, and calcification has been associated with the early and late stage of OA [13]. The inhibition of chondrocyte hypertrophic differentiation was considered as a therapeutic target for OA progression. The Ddr2, a receptor tyrosine kinase, that can be activated by degrade type II collagen has been reported to play a role in chondrocyte differentiation in OA. The increased expression of Ddr2 was reported to be associated with OA progression and elevated MMP13 expression in the surgically induced OA in mice [14]. Moreover, Ddr2 modulated Runx2 activity and stimulated the expression of Col X in hypertrophic chondrocytes (ADTC5 cells) [15]. The role of Ddr1 in chondrocyte hypertrophic differentiation is still not clear. Col X and MMP13 were the mostly widely used markers for hypertrophic chondrocytes [13]. Apoptosis is also an important marker for detecting hypertrophic chondrocytes in late staged OA cartilage. The TUNEL staining of late-staged human OA cartilage showed that the chondrocytes in the upper zone of cartilage undergo terminal differentiation and eventually die by apoptosis [16]. In the current study, 7 rh treatment significantly decreased the expression of Col X and MMP13 and also reduced chondrocyte apoptosis by decreasing the expression of activated caspase 3 in OA cartilage. These findings suggested that the IA injection of 7 rh reduced chondrocyte hypertrophic differentiation and apoptosis in the OA cartilage, thereby reducing OA progression.

Autophagy is an essential process in maintaining cellular metabolism and homeostasis. Emerging evidence suggests that the deregulation of autophagy is a crucial factor in the pathogenesis of OA [17,18]. Caramés, B. et al. studied the expression of autophagy regulator and chondrocyte apoptosis in the articular cartilage of human OA and the experimental OA, and the authors demonstrated that the autophagy regulators were reduced in the human OA and the aging-related or surgically induced OA in mice accompanied by an increase in chondrocyte apoptosis [17]. Recently, the same researchers used green fluorescent protein–light chain transgenic mice to detect autophagy activation in normal and aging cartilage and indicated that the autophagy regulators were reduced before the onset of cartilage degradation and the decrease in chondrocyte cellularity [18]. The mTOR signaling pathway plays a central role in regulating the initiation, processing, and termination of autophagy. The cartilage-specific deletion of mTOR upregulated autophagy and protected mice from OA [10]. In addition, autophagy activated by rapamycin (an inhibitor of mTOR) reduced the severity of experimental OA [19]. Similar to previous studies, our results showed significantly increased mTOR expression after experimental OA induction, indicating that the reduction in the autophagy process leads to articular cartilage degeneration [11,19]. Furthermore, we also found that beclin1 and LC3, the major indicators for autophagosome formation, were expressed in normal cartilage, indicating a functional autophagy process. However, the expression of autophagy markers (beclin1 and LC3) was significantly suppressed after ACLT, implying insufficient autophagy in OA cartilage. These findings indicated that articular cartilage degradation after ACLT was related to autophagy dysfunction. In this study, our results showed that the IA of 7 rh significantly restored autophagy function and further reduced the progression of OA, as shown by the reduced immunostaining of activated mTOR, the increased immunostaining of beclin1 and LC3, and histologically lower GAG loss and a lower OARSI score. Based on these findings, we suggested that the reduction in chondrocyte apoptosis, mediated by the activation of autophagy, may be a part of the mechanism of action of 7 rh on preventing OA progression.

In conclusion, our results showed that the IA injection of 7 rh reduced cartilage degradation in ACLT-induced OA animals. The inhibition of Ddr1 can reduce chondrocyte hypertrophic differentiation and chondrocyte apoptosis in the OA cartilage, as well as recover the autophagy function that was impaired by OA. We demonstrated that the inhibition of Ddr1 in articular cartilage could modulate OA progression and prevent chondrocyte apoptosis by promoting autophagy in OA cartilage. These findings suggested that 7 rh may be a potential disease-modifying drug to prevention OA progression.

## 4. Materials and Methods 

### 4.1. Human Articular Chondrocyte (HAC) Cultures

The human articular chondrocyte (HAC) cell line was purchased from Lonza Walkersville Inc. (8F3339, LONZA, MD, USA). The HAC cells were cultured in Dulbecco Modified Eagle Medium (DMEM) containing 10% fetal bovine serum (FBS), 100 IU/mL penicillin, and 100 μg/mL streptomycin at 37 °C in a humidified atmosphere of 5% CO_2_.

### 4.2. Cell Viability Assay

Cell viability was measured using Counting Kit-8 (CCK-8 assay). A total of 10,000 HAC cells per well were cultured in 96-wells cell culture plate and then incubated for 48 h at 37 °C. After that, HAC cells were cultured in 200 μL culture medium with 7 rh (6.9 nM or 13.8 nM) or co-cultured with 7 rh and IL-1β (10 ng/mL) in 5% CO2 for 3h. Then, 10 μL of CCK-8 reagent (E-CK-A361, Elabscience, Houston, TX, USA) was added and the optical density at 450 nm was measured using a multifunction microplate reader (Synergy H1, BioTek, Winooski, VT, USA) after incubation for 2 h at 37 °C.

### 4.3. Animals Experiments

All animal experiments were performed following the approval of the Institutional Animal Care and Use Committee-IACUC from Kaohsiung Medical University (The project identification code and date: IACUC105127; 1 Aug 2017–31 July 2020). A total of 40 healthy male C57BL/6 mice (8-weeks-old) were obtained from the Jackson laboratory (National laboratory animal center, Taiwan), and were housed under the standard conditions in Animal Center of Kaohsiung Medical University (Kaohsiung, Taiwan). The animals were randomly assigned into four groups, including the sham group (sham operation treated by Normal saline, *n* = 10), ACLT group (OA induction treated by Normal saline, *n* = 10), ACLT plus 7 rh 6.9 nM (*n* = 10), and ACLT plus 7 rh 13.8 nM (*n* = 10). OA was surgically induced in 8-weeks-old C57BL/6 mice by ACLT in the right knee. According to study design, the mice either received IA injection with the same volume (10 μL) of Normal saline, 7 rh 6.8 nM, or 7 rh 13.8 nM three times per week from 7 to 9 weeks-old and then one dose per week until sacrifice at 13 weeks-old. 

### 4.4. Weight Bearing Distribution Test

The weight-bearing ability after OA-induction was measured by Dual Channel Weight Averages (Singa Technology Corporation, Taiwan), which can analyze the weight-bearing of each hind paw. The changes in joint weight-bearing capacity represent the severity of the experimental joint symptoms. The weight-bearing test was performed at one week before ACLT and then every week until the mice were euthanized.

### 4.5. Running Endurance

Mice were subjected to run on a Columbus Instruments rodent treadmill (Columbus, OH, USA) once a week before and after OA-induction until the mice were euthanized. The running speed started from 8.5 m/min and lasted for 3 min. Thereafter, the treadmill speed increased by 2.5 m/min every 3 min with a treadmill angle of 5 degrees until the maximum running speed was 25 m/min. The limit of running endurance recording time was 15 min, and the experiment stopped after reaching the maximum duration. During the whole process, a mild electric shock caused an uncomfortable shock but not physical damage to the mice and was set according to the previous study [17].

### 4.6. Histopathological Assessments

The mice tibia samples were fixed in 10% buffered paraformaldehyde for 2 days and then decalcified with buffered ethylenediaminetetraacetic acid (0.5 M, pH 7.4). After dehydration and embedding in paraffin, the tissue blocks were cut in coronal with a thickness of 5 μm. Glycosaminoglycan (GAG) was stained by Safranin O-Fast Green (1% safranin O counterstained, 0.75% hematoxylin, and then 1% Fast Green; Sigma, St. Louis, MO, USA) and was used to evaluate the severity of OA. All of the histological results were assessed by two independent researchers blinded to any other information according to the histology grading system of Osteoarthritis Research Society International (OARSI) [18].

### 4.7. Immunohistochemistry (IHC) for Type X Collagen (Col X), Indian Hedgehog (IHH), Matrix Metalloproteinases 13 (MMP13), Caspase 3, mTOR, Light Chain 3 (LC3), Beclin-1

Tissue sections were deparaffinized, rehydrated and then blocked with 3% hydrogen peroxide. The samples were prepared for indirect immune detection by mouse- and rabbit-specific horseradish peroxidase/diaminobenzidine detection kit (ab64264, Abcam, Cambridge, MA, USA) following the manufacturer’s instructions. The primary antibodies used in this study were rabbit polyclonal antibodies to Col X (ab58632, Abcam, Cambridge, MA, USA), IHH (ab52919, Abcam, Cambridge, MA, USA), MMP13 (ab39012, Abcam, Cambridge, MA, USA), activated caspase-3 (ab2302, Abcam, Cambridge, MA, USA), phosphorylated-mTOR(Ser235/236)(4858s, Cell Signaling, Danvers, MA, USA), LC3 (14600-1-AP, Proteintech, Rosemont, IL, USA), and beclin 1 (11306-1-AP, Proteintech, Rosemont, IL, USA). The sections were counterstained with hematoxylin and the immunolocalized nuclei were stained in brown. For quantification, the sections of related target proteins staining were digitalized at 400 times magnification in a total of 2560 × 1920 pixel and 300 dpi digital images in JPG file format using TissueFAXS microscope (TissueGnostics GmbH, Vienna, Austria). The digital images were analyzed to quantify the total amount of related target proteins staining using HistoQuest (TissueGnostics, Los Angeles, CA, USA) analysis software. Total area of related target protein staining was detected after automatic color separation by HistoQuest. The staining intensity was measured as mean intensity of all pixels with an automatic background threshold range from 5 to 255. The results were given as percentage per mm^2^ of total tissue area. 

### 4.8. Terminal Deoxynucleotidyl Transferase dUTP Nick End Labeling (TUNEL) Assay 

TUNEL assay was performed on proximal tibia tissue sections to detect chondrocyte apoptosis (12156792910, Roche, Branchburg, NJ, USA). The percentages of TUNEL-positive cells in chondrocytes relative to 4′,6-diamidino-2-phenylindole (DAPI)-stained cells were calculated and an analysis was conducted using a Leica immunofluorescence system (Leica MicroImaging). Photographs from three independent experiments were conducted and calculated for each experimental group.

### 4.9. Statistical Analysis

All data were presented as mean±SEM. The results were analyzed using one-way ANOVA, and multiple comparisons were conducted by Tukey’s HSD using GraphPad Prism (version 5.0). The statistical significance was considered as *p* < 0.05.
